# Strengthening public health pesticide management in countries endemic with malaria or other major vector-borne diseases: an evaluation of three strategies

**DOI:** 10.1186/1475-2875-13-368

**Published:** 2014-09-18

**Authors:** Henk van den Berg, Rajpal S Yadav, Morteza Zaim

**Affiliations:** Laboratory of Entomology, Wageningen University, PO Box 8031, Wageningen, 6700 EH the Netherlands; Vector Ecology and Management, Department of Control of Neglected Tropical Diseases, World Health Organization, Geneva, Switzerland

## Abstract

**Background:**

Public health pesticides has been the mainstay control of vectors of malaria and other diseases, and public health pests, but there is increasing concern over how these pesticides are being managed. Poor pesticide management could lead to risks to human health and the environment, or diminish the effectiveness of interventions. Strategies for strengthening the management of public health pesticides, from manufacture to disposal, should be evaluated to propose future directions.

**Methods:**

The process and outcomes of three strategies were studied in five regions of the WHO (African Region, Eastern Mediterranean Region, South-East Asia Region, Western Pacific Region, and American Region) and 13 selected countries. These strategies are: regional policy development, in-depth country support and thematic support across countries.

**Results:**

Consensus, frameworks and action plans on public health pesticide management were developed at regional level. Country support for situation analysis and national action planning highlighted weaknesses over the entire spectrum of pesticide management practices, mainly related to malaria control. The thematic support on pesticide quality control contributed to structural improvements on a priority issue for malaria control across countries.

**Conclusions:**

The three strategies showed promising and complementary results, but guidelines and tools for implementation of the strategies should be further improved. Increased national and international priority should be given to support the development of policy, legislation and capacity that are necessary for sound management of public health pesticides.

## Background

Malaria and other vector-borne diseases, notably dengue, lymphatic filariasis, leishmaniasis, and Chagas disease, together with domestic pests, continue to be a major problem for global public health [1], causing immense suffering and preventing people from escaping poverty. Vector control, aiming to reduce vector populations or vector-human contact, plays a major role in the prevention, control or elimination of malaria and most other vector-borne diseases [2, 3].

The use of public health pesticides (i.e. vector control pesticides, household insecticides, and professional pest management pesticides [4]) has been the mainstay control of vectors of malaria and other diseases, and other pests, in or around human habitation [5, 6]. However, there is increasing concern over how these pesticides are being managed, particularly in resource-poor countries. Poor pesticide management could pose risks to human health and the environment, or reduce the effectiveness of interventions. Recent reports have indicated an unregulated trade in substandard pesticide products [7, 8], rapid development of insecticide resistance in malaria vector populations [9–11], and the existence of large stockpiles of obsolete vector control insecticides [12]. Particularly, the scaling-up of vector control interventions such as indoor residual spraying and long-lasting insecticidal nets for malaria control have, in many countries, led to insecticide resistance, leaving programmes with a reduced number of available insecticide options. An additional problem is that the capacity for evidence-based decision making on vector control interventions is weak in many countries, leading to suboptimal interventions and waste of valuable resources [13, 14].

Integrated vector management, defined as a rational decision-making process for the optimal use of resources for vector control, is being promoted by the World Health Organization (WHO) as the preferred approach for increasing the efficiency, effectiveness, sustainability, and environmental soundness of vector control [15, 16]. Several examples of IVM exist [13, 17–19]. The integrated vector management approach applies equally to the control of domestic pests. As insecticides have a dominant role in current strategies of vector and pest control, there is a significant overlap between pesticide management and integrated vector management. Hence, improvement in how insecticides are being selected, used and evaluated will have immediate benefits for the control of vectors and pests. This elementary role of pesticide management for achieving health outcomes (e.g. reduction in pesticide poisoning, reduction in malaria incidence) deserves increased attention in advocacy at national and international level.

The International Code of Conduct on Pesticide Management (referred to as: Code of Conduct) provides a voluntary and globally accepted standard for effective life-cycle management of agricultural and public health pesticides [20]. The life-cycle concept of pesticide management refers to the legislation, regulatory control and practices related to all stages of a pesticide’s life, from manufacture and import to use and disposal. However, a global survey has indicated that the basic standards, rules and practices needed to effectively and safely manage public health pesticides are not in place in a large segment of vector-borne disease-endemic countries [21, 22]. Low priority given to capacity building and regulation on pesticide management has been attributed to the lack of awareness of policy makers and advisors about the risks of pesticides [23]. This underscores the need for awareness raising and capacity building at country level. A clear gap in implementation of the Code of Conduct was demonstrated between higher and lower income countries, with lower income countries lagging behind considerably [24].

In recent years, there has been increased global support for policy and programmes on pesticide management. In 2010, the World Health Assembly adopted Resolution WHA 63.26, which urges Member States, *inter alia*, to adopt or strengthen sound national policies and legislation on safe handling and disposal of obsolete pesticides and to establish or strengthen capacity for the regulation of the sound management of pesticides throughout their life cycle [25]. Moreover, the Bill & Melinda Gates Foundation (BMGF) funded a global initiative dedicated to improve the management of public health pesticides [26].

The objective of this paper is to examine three available strategies for strengthening the management of public health pesticides, review their outcomes and propose future directions. As far as known to the authors, no previous studies have addressed this topic.

## Methods

**Figure 1:**
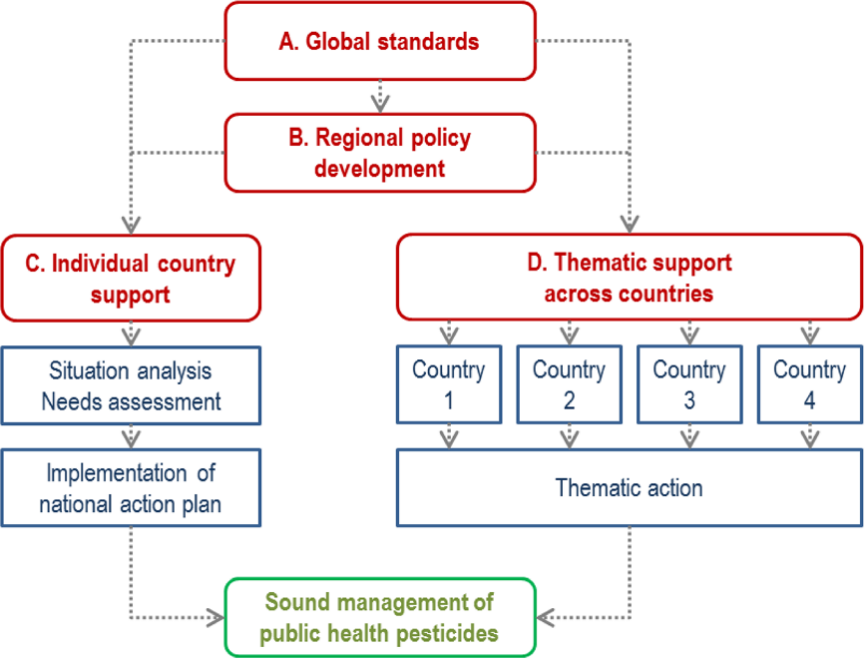
**Strategies for strengthening pesticide management.** Diagram showing four strategies, indicated as **A**-**D**.

Several strategies are presented for strengthening public health pesticide management (Figure 1). At the global level, support is needed on internationally agreed standards, which include guidelines and pesticide specifications; this strategy which is a core mandate of the WHO Pesticide Evaluation Scheme is presented in a separate contribution (van den Berg H, Yadav RS, Zaim M: Setting international standards for the management of public health pesticides: where are we now?, submitted). The international standards are needed to assist individual countries in their legislation, regulation and practices of pesticide management. This paper concentrates on strategies at regional level and country level.

Strategies of regional policy development, individual country support, and thematic support across countries, had been developed by WHO in preparation of the BMGF project, based on the outputs of previous consultation meetings and based on the results of an earlier survey [27]. Workable strategies other than those presented here are not known to the authors.

### Regional policy development

Policy development at the regional level is a strategy aiming to raise awareness and political commitment on public health pesticide management among countries. A regional policy has potential to influence policy development in a large number of countries. In accordance with the recommendations under Resolution WHA 63.26 [25], consultation workshops were organized by the WHO in its American, African, Eastern Mediterranean, Southeast Asia and Western Pacific Regions, with member-country representation, in order to raise awareness, explore challenges, barriers and opportunities, build consensus, and develop regional policy on public health pesticide management. The workshops followed a process of reviewing the results of the aforementioned global survey on public health pesticide management, identification of the challenges to pesticide management in the region, and development of a consolidated document to guide the improvement of pesticide management in the region, particularly related to malaria vector control.

Out of the 142 countries recognized as being endemic with or at risk of major vector-borne diseases [28], 12 priority countries were selected by WHO for capacity strengthening activities under the BMGF Project: Cameroon, Gambia, Kenya, Madagascar, Mozambique, Tanzania (WHO African Region), Morocco, Sudan (WHO Eastern Mediterranean Region), Thailand (WHO Southeast Asia Region), Cambodia (WHO Western Pacific Region), Ecuador, and Guatemala (WHO American Region). The selection of countries was based on criteria of vector-borne disease burden, amounts of insecticide used, and commitment to reinforce pesticide management capacity. As 13th country, Oman government officials participated in the activities using their own financial resources.

At country level, two separate strategies were identified: in-depth support for situation analysis and action planning according to the individual country’s context; and thematic support provided across a number of countries.

### Individual country support

Country-level situation analysis of the existing legal, institutional, technical, and administrative conditions was selected as strategy because it was seen as first step towards developing a national action plan for improving public health pesticide management. In the period 2008-11, each of the 13 selected countries, which are indicated above, was assisted by WHO in conducting a comprehensive assessment on their situation on public health pesticides, using existing methods on situation analysis [29]. Briefly, the methods involved the gathering and analysing of information in response to detailed questions on a range of components, including legislative control, legal instruments, pesticide use, pesticide management practices, participation in international conventions, and financial resources for pesticide management. The Code of Conduct and WHO guidelines on pesticide management [4] were used as reference documents to assist in the identification of shortfalls.

To carry out a situation analysis, each country established a task force with representatives from the main implicated ministries. This task force conducted workshops with stakeholders from other ministries, civil society and the private sector to solicit inputs and prioritize a number of key issues as topics to be addressed in national action plans. Documentation of the situation analysis was performed by national experts.

### Thematic support across countries

For certain aspects of pesticide management that are a known problem in various countries, a strategy was used for providing thematic support across countries. Previous consultation meetings organized by WHO had singled out pesticide quality control as a priority issue in pesticide management that could practicably be addressed by standardized capacity building activities across countries. Substandard, counterfeit and adulterated pesticides, are of major concern to developing countries, posing safety risks and undermining effectiveness of malaria control interventions. Data on the quality of traded products are mostly absent because countries generally lack the equipment, facilities and training for pesticide quality control [21, 22].

In the period 2009-2011, support was given to 12 selected countries (Oman did not meet the selection criteria) by means of coordinated inputs from international experts with use of training methods and training courses. In-country chemical laboratories were assessed for their capacity for pesticide quality control, or for their role as national reference laboratory, using existing criteria [30].

## Results

### Regional policy development

The regional consultations have highlighted the urgency of, and strengthened consensus building on the development of regional policies or action plans on sound pesticide management, with main focus on malaria control. As outcomes of these consultations, frameworks and action plans have been developed in several WHO regions, with objectives addressing legislation, registration, management, quality control, and application (Table 1). These results have been shared with relevant WHO advisory committees for development of priority actions.

**Table 1:** **Outcomes of regional policy consultations**

	WHO Region				
Item	African	Americas	Eastern Mediterranean	Southeast Asia	Western Pacific
Type of outcome	Guidelines; Meeting consensus [31]	Meeting consensus [32]	Regional resolution; Regional framework [33, 34]	Guidelines [35]	Regional framework [36]
Year developed	2011	2011	2012	2010	2012
Title	Guidelines on public health pesticide management policy for the WHO African Region; complemented by workshop outputs	Antigua Charter for the sound management of pesticides in public health	Framework for action on the sound management of public health pesticides (2012-2016)	Guidelines on public health pesticide management policy*	Draft Regional framework for action on the sound management of public health pesticides (2012-2016)
Objectives/recommendations:					
a. Legislation	Review/revise pesticide legislation	Strengthen legal framework and policy development	Develop comprehensive pesticide policy and legislation	-	Develop comprehensive pesticide policy and legislation
b. Registration	Strengthen capacity for registration and post-registration monitoring	Monitor and report on registration	Operate an effective pesticide registration scheme	-	Operate an effective pesticide registration scheme
c. Management practices	Develop capacity for procurement, transport, distribution, storage, and disposal	Improve storage, disposal, exposure reporting, product traceability, and alternative products	Improve procurement, storage, distribution and disposal	-	Improve procurement, storage, distribution and disposal
d. Quality control	Strengthen laboratories, forge inter-country collaboration	Monitor and report on selection, procurement, and quality control	Improve quality control, post-registration monitoring and enforcement	-	Improve quality control, post-registration monitoring and enforcement
e. Application	Build capacity for monitoring and evaluation of vector control operations	Training on safe use and application of pesticides	Ensure safe and judicious application of pesticides	-	Ensure safe and judicious application of pesticides
f. Efficacy testing	-	Monitor pesticide efficacy and resistance	-	-	-
e. Regional cooperation	-	Improve harmonization of regulations, information sharing	-	-	-

*Guidelines for countries, without specific objectives or recommendations.

In the Eastern Mediterranean Region, the consultations have led to the development of a regional resolution on managing the use of public health pesticides, which was adopted in October 2011 by the WHO Regional Committee [37]. In turn, the resolution resulted in the development of a regional framework for action on the sound management of public health pesticides [38]. In the American Region, a consultation with wide representation from member countries led to a consensus on recommendations, called the ‘Antigua Charter’ [32]. In the Western Pacific Region, a Regional Framework for Action has been developed [39].

In the African and Southeast Asia Regions, a different approach was taken. Instead of developing a regional policy or resolution, regional guidelines were prepared on the development of public health pesticide policy [31, 35]. This was conducted through regional consultation meetings. The objective was to assist in policy development at country level.

Collectively, the outcomes of the regional consultations have demonstrated the interest and willingness among country representatives to tackle the problems encountered in the life-cycle management of public health pesticides. The impact of these regional outcomes in terms of policy development and political commitment at country level should be tracked in the years ahead. As a first spin-off of the activities in the African Region, national policies on public health pesticide management in support of malaria control have recently been developed in the Gambia, Madagascar, Mali, and Sierra Leone.

### Individual country support

The results of the situation analysis from the 13 selected countries showed shortcomings and gaps in pesticide management over the entire spectrum of pesticide management practices, with considerable differences between countries. Most countries had inadequate legislation on procurement, transport, storage and disposal of public health pesticides. A number of countries did not have appropriate stock management capacity, storage facilities or disposal mechanisms for public health pesticides, and lacked guidelines on procurement, transport, storage, use, and waste disposal of public health pesticides. Countries reported inadequate capacity or guidelines for assessment of registration dossiers on public health pesticides. Only half of the countries reported having a fully operational, quality control laboratory to analyse public health pesticides. Moreover, countries reported poor intersectoral coordination on public health pesticides, and a lack, or inadequate implementation, of national policy on integrated vector management and pesticide management.

**Figure 2:**
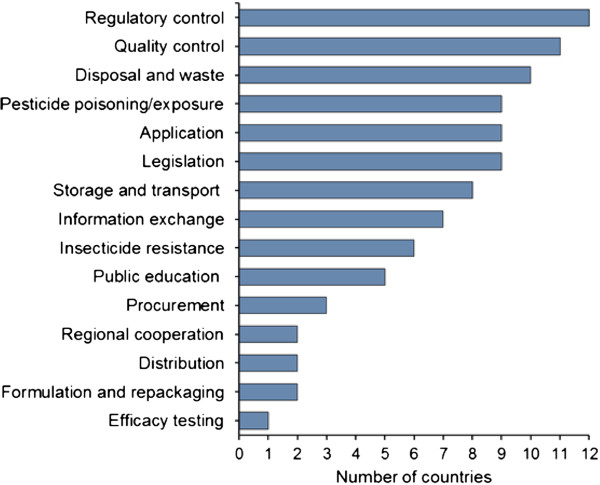
**Country priorities.** Main topics occurring in national action plans on public health pesticide management of twelve countries (data from all but one of the selected countries).

The outputs of the situation analysis were subsequently used by countries to identify requirements and to develop a national action plan to strengthen the management of public health pesticides. Each national action plan incorporated a number of key topics (eight on average). Topics most commonly selected by countries were: regulatory control, pesticide quality control, disposal and waste, monitoring of pesticide poisoning or exposure, use application, legislation, and storage and transport (Figure 2). Pesticide quality control was selected as key topic in 11 out of 12 countries, which justifies the selection of this topic for thematic support across countries. Budgets attached to national action plans ranged from US$0.01 m-2.7 m per country (median US$1.04 m), spread over one or two years. It is unlikely that these funds, much of which was needed for training and equipment, will be available from national sources. Hence, most countries will require additional support for implementation of their action plan in support of their programmes on malaria control or elimination.

### Thematic support across countries

Thematic support provided to 12 countries has highlighted the primary weaknesses on the topic of pesticide quality control and contributed to human resources development on pesticide specification. To meet the standards set by the Food and Agriculture Organization (FAO) and WHO, pesticide quality control should comprise the identification of active ingredient and determination of the content of active ingredient; determination of physical-chemical properties of pesticide formulations; and determination of levels of relevant impurities [40]. The assessments in the 12 countries revealed that functional laboratories for pesticide quality control were few. The existing laboratories showed various deficiencies, whereas national reference laboratories still needed to be established in many countries. Most of the existing laboratories were capable to perform basic tests, e.g., on active ingredients, but not to perform work on physical-chemical properties or on relevant impurities. The assessments pinpointed where improvements will be needed in terms of equipment, infrastructure, quality assurance systems, and regular training to enable full quality control of pesticides in countries according to FAO/WHO specifications.

A major problem is that in a number of countries, quality control laboratories are concentrating on agricultural pesticides and do not include public health pesticides. This problem is compounded by the fact that pesticide legislation in some countries does not include public health pesticides [21]. Quality control schemes are very similar for all pesticides, irrespective of their end use. Therefore, collaboration on quality control and legislation between sectors of health and agriculture should be established to make efficient use of available national resources.

To support the selected countries in their establishment of national quality standards for public health pesticides, three-day training courses on the use of FAO/WHO specifications for public health pesticides were carried out in each of the 12 countries for registration authorities and chemical laboratory staff [41]. The curriculum with group exercises covered specifications for technical grade active ingredients, formulations, relevant impurities, and determination of equivalence, using the FAO/WHO training manual on specifications [41].

The thematic support on pesticide quality control has made an important step towards structural improvements across 12 countries, through assessment of laboratories and training on pesticide specifications. Nevertheless, there is still a long way to go in human resources development in the selected countries, for example, through on-the-job training on the actual laboratory procedures. Also, complex laboratory work, such as the determination of equivalence of technical grade active ingredients, will require cooperation with specialist chemists and toxicologists who should establish the impurity profiles or evaluate hazard profiles of the products. Such expertise may be lacking or inadequate in most countries. To increase the prospects for improvement, it has been suggested that laboratories should link or collaborate with other laboratories, for example through the global network of the Collaborative International Pesticides Analytical Council [42]. Moreover, for small or resource-poor countries, it could be preferable to establish laboratory capacity at sub-regional or regional level, so as to make more efficient use of available resources and expertise through work sharing between countries [21]. As an example, in some regions there is high participation of countries in regional pesticide registration schemes, such as the Central Africa Inter-State Pesticides Committee [43].

## Discussion

The shortcomings in public health pesticide management as identified by the 13 selected countries are most likely undermining the effectiveness and safety of pesticides. Moreover, previous results have indicated that the shortcomings are widespread across malaria-endemic countries and regions [21, 22]. It will be a major challenge for countries to strengthen their policy, legislation and capacity necessary to enable the implementation of the existing global standards on public health pesticide management. The presented strategies of regional policy development, in-depth country support, and thematic support across countries, provide valuable lessons for reinforcing and expanding the international support to countries in need. The support should be provided within the context of IVM, where pesticides are not seen as the only option for vector control [15]. The three strategies are expected to increase capacity needed for IVM by improving country-level decisions on selection and use of vector control insecticides.

The strategy of regional policy development resulted in consensus building and/or policy statements among country representatives in five WHO regions. The outcomes were most advanced in the Eastern Mediterranean Region, where a resolution and a regional action plan were adopted. In general, the regional consultations have raised awareness among country representatives about the urgency of sound public health pesticide management. Moreover, the published frameworks, guidelines and resolutions are expected to uplift the issue on the national political agenda. The regions took different approaches to strengthening policy on public health pesticide management, either to develop a regional policy (with or without framework) or guidelines for policy making at country level. It would seem appropriate that regional policy is accompanied by a guidelines document, and vice versa, in order to raise pesticide management on the regional agenda while also providing the methods to facilitate policy development at country level.

Clearly, regional policy development should not be a stand-alone activity but must be supplemented by country support, lest the regional policy may not be adopted and implemented. In turn, it is expected that country support for pesticide management will be more fruitful if endorsed by a regional policy. Continued monitoring on the implementation of regional frameworks and action plans by individual countries is required in the years ahead.

The multifaceted situation, with various shortcomings in pesticide life-cycle management per country and with context-specific differences between countries, demands a systematic and well-targeted approach to supporting individual countries. In this respect, the strategies of in-depth country support and thematic support across countries appeared to be complementary, by addressing each country’s context while also prioritizing common problems among countries. The in-depth support for situation analysis and action planning according to each country’s unique context of legislative, institutional and biological factors has demonstrated to be a vital starting point, but demands considerable investment of time and energy. The exercise contributed to an increased awareness among key stakeholders about the deficiencies in pesticide management. However, the large number of shortcomings identified by individual countries resulted in the development of rather ambitious and unfocused national action plans that would be difficult to accomplish unless further priority setting is conducted. Consequently, the existing methods on situation analysis need revision to provide clearer indicators for use in situation analysis and tools to aid in prioritization, needs assessment and action planning [29]. Countries should be supported, where needed, in the development of proposals for mobilization of resources.

The results of thematic support on pesticide quality control demonstrated how a prioritized issue can be efficiently and effectively addressed across a number of countries to achieve tangible results. This strategy depends largely on the availability of standard methods and tools and on international expert inputs. As a strategy, however, the thematic support could be further strengthened, for example, by setting criteria for the selection of themes and countries, and by conducting feasibility studies at baseline.

Both country-level strategies (in-depth support and thematic support) will present well-defined options for donor funding and programmatic response. For example, thematic support of West African countries on insecticide resistance monitoring of malaria vectors will constitute a viable package for external funding. Alternatively, disposal of obsolete pesticides could be efficiently implemented across countries, following the example of the Africa Stockpiles Programme [44]. At individual country level, in-depth analysis and planning on pesticide management could be appropriate as stand-alone project or for incorporation into new or ongoing development programmes. Also, countries should require suppliers or manufacturers of pesticides to contribute towards sound pesticide management of their products, for example, by stipulating stewardship support for quality control in public tenders for procurement of pesticides.

Every strategy should emphasize a multisectoral approach, because many pesticides are used in more than one sector, and the life-cycle management of a pesticide generally involves the actions of several sectors. Also, it is important to bear in mind that the global insecticide use for vector control constitutes is only a minor fraction of the global consumption of insecticides (1.5% of total) or pesticides (0.3% of total) [6, 45]. Household insecticides are estimated to take up between 5 and 10% of the total global insecticide market [46]. The lion’s share of all pesticides is for use in agriculture.

Intersectoral coordination should aim to harmonize legislation and practices across sectors. The experiences from the 13 selected countries showed that coordination between sectors was weak at baseline but recent task forces and workshops brought together the stakeholders from several sectors to share information, conduct situation analyses and develop common action plans on pesticide management. Future work should capitalize on these achievements. In line with a multisectoral approach, the external support provided at global and country level should be coordinated between specialized international organizations. Several organizations, notably WHO and FAO, together with industry and research institutions, have recently strengthened their collaboration at global level through the development of guidelines and standards on pesticide management (van den Berg H, Yadav RS, Zaim M: Setting international standards for the management of public health pesticides: where are we now?, submitted). However, the international organizations need to improve their coordination and collaboration in their support on pesticide management to individual countries in need.

Thus far, the WHO has taken a lead in providing country support on public health pesticide management in collaboration with FAO [26]. Indeed, several components of public health pesticide management are specific to the health sector and are commonly implemented by disease control programmes. This refers to pesticide procurement, application and monitoring of insecticide resistance. However, the other components of public health pesticide management overlap with those of pesticides used in other sectors, predominantly agriculture, in a number of ways.

In most countries, legislation covers all pesticides. Also, registration is commonly conducted by one central authority, in accordance with the Code of Conduct [8, 20]. Likewise, quality control facilities generally cover all pesticides, to make efficient use of available resources. Nevertheless, a number of countries still have legislation that does not cover public health pesticides; have more than one registration authorities; or have pesticide quality control that does not include public health products [21]. Several other components of pesticide management that would ideally cover all pesticides are: monitoring of exposure and poisoning, disposal, public education, information exchange and Regional cooperation.

Considering the tasks ahead, future support for capacity building at country level should be coordinated and shared between the international agencies, noting their technical capacity and resources. WHO, FAO and UNEP should intensify their collaboration in providing policy options, shaping research agendas and providing international technical support in the life-cycle management of pesticides.

## Conclusions

Weaknesses in the management of public health pesticides are widespread in countries endemic with malaria or other vector-borne diseases, and will adversely influence the effectiveness and safety of how the pesticides are used, distributed, stored, or disposed. Therefore, increased priority must be given by governments, international agencies and donors to invest in strategies that support countries in the development of policy, legislation and capacity necessary for sound management practices for public health pesticides. Future support should build on the available evidence and recent achievements to achieve tangible improvements in public health pesticide management where most needed.

The study results indicated that regional policy development, in-depth country support for situation analysis, and thematic support across countries are three promising and largely complementary strategies for strengthening public health pesticide management. Nevertheless, the methods and tools used in these strategies need further improvements. Each strategy should emphasize a multisectoral approach aiming to harmonize legislation and practices across sectors. Accordingly, specialized international organizations, notably FAO, UNEP and WHO, should coordinate and collaborate in providing country-level support.
